# A tutorial in displaying mass spectrometry-based proteomic data using heat maps

**DOI:** 10.1186/1471-2105-13-S16-S10

**Published:** 2012-11-05

**Authors:** Melissa Key

**Affiliations:** 1 Center for Computational Diagnostics, IU School of Medicine, Indianapolis, IN, USA

## Abstract

Data visualization plays a critical role in interpreting experimental results of proteomic experiments. Heat maps are particularly useful for this task, as they allow us to find quantitative patterns across proteins and biological samples simultaneously. The quality of a heat map can be vastly improved by understanding the options available to display and organize the data in the heat map.

This tutorial illustrates how to optimize heat maps for proteomics data by incorporating known characteristics of the data into the image. First, the concepts used to guide the creating of heat maps are demonstrated. Then, these concepts are applied to two types of analysis: visualizing spectral features across biological samples, and presenting the results of tests of statistical significance. For all examples we provide details of computer code in the open-source statistical programming language R, which can be used for biologists and clinicians with little statistical background.

Heat maps are a useful tool for presenting quantitative proteomic data organized in a matrix format. Understanding and optimizing the parameters used to create the heat map can vastly improve both the appearance and the interoperation of heat map data.

## Background

Heat maps are an efficient method of visualizing complex data sets organized as matrices. In a biological context, a typical matrix is created by arranging the data such that each column contains the data from a single sample and each row corresponds to a single feature (e.g. a spectrum, peptide, or protein).

Correlation and interaction matrices are also common [1,2], but anything which can be arranged as a matrix can be displayed, for example the results from a series of statistical tests. In each case, the power of using a heat map is its ability to display patterns in large quantities of data without summarizing.

A heat map performs two actions on a matrix. First, it reorders the rows and columns so that rows (and columns) with similar profiles are closer to one another, causing these profiles to be more visible to the eye. Second, each entry in the data matrix is displayed as a color, making it possible to view the patterns graphically. Multiple methods exist to accomplish these two tasks. The purpose of this tutorial is to demonstrate how these methods can be optimized for specific types of matrices. To accomplish this, we describe a few common methods in detail, and demonstrate how these methods are implemented in the open-source statistical programming language R [3]. Then, we use these methods to analyze the Prostate2000Peaks data set, which is available in R through the msProstate package [4]. Specifically, we show (1) what steps are necessary to prepare the example data set for display in a heat map, (2) how different methods compare in showing clusters of features and spectra, and (3) how to map colors to significance results in a systematic manner that is easily interpretable. Table 1 lists four independently maintained packages available in R contain heat map functions. Most examples in this tutorial are created using the heatmap.2 function in the gplots package [5], which is the most customizable of the heat map packages considered. For basic usage, the heatmap function is automatically installed and loaded as part of the stats package in R. The function heatmap.plus, available in the package heatmap.plus[6], can display multivariate group information and has slightly improved layout controls. The functions heatmap_2 and heatmap_plus are both part of the Heatplus package [7] in Bioconductor [8], and are located in Bioconductor repositories although they do not require Bioconductor to run. These functions are based on an ancient version of the standard heatmap function, and as such, they are the most different from the other functions - with some drawbacks as a result. That said, they also contain unique features not present in the other functions. A more detailed description of the features of each function and all code used in this tutorial are included in Additional Files 1, 2.

**Table 1:** Heat map functions.

Function	Package	Version
heatmap	stats [3]	R version 2.12.1
heatmap.2	gplots [6]	2.8.0
heatmap.plus	heatmap.plus [5]	1.13
heatmap_2	Heatplus [7]	1.16.0
heatmap_plus	Heatplus [7]	1.16.0

The heat map functions described in this tutorial.

## Heat map components

A heat map is the combination of two independent procedures applied to a data matrix. The first procedure reorders the columns and rows of the data in order to make patterns more visible to the eye. The second procedure translates a numerical matrix into a color image. Here, we use a series of illustrative examples to introduce the concepts from each procedure, and show how they impact the final heat map.

## Data reordering

Data reordering plays a critical role in demonstrating patterns in the data. The goal of data reordering is to place columns (or rows) with similar profiles near one another so that shared profiles become more visible. Most heat maps use an agglomerative hierarchical clustering algorithm to group the data, and display this information using a dendrogram. An agglomerative hierarchical clustering algorithm on *n *objects begins by considering each object to be a group of size 1. At each step, the two closest groups are merged together, until all *n *objects are in a single group.

A dendrogram is a common method of graphically displaying the output of hierarchical clustering. At the bottom, each line corresponds to each object (clusters of size 1). When two clusters are merged, a line is drawn connecting the two clusters at a height corresponding to how similar the clusters are. The order of the objects is chosen to ensure that at the point where two clusters are merged, no other clusters are between them, but this ordering is not unique. When two clusters are merged, the choice of which cluster is on the left and which is on the right is arbitrary.

Inherent to this procedure is the ability to measure the similarity between clusters, that is to represent similarity with a measurement of distance. In fact, many hierarchical clustering algorithms only look at distances between data points, never at the original data. Two types of distance measurements are important: the distance between individual observations (distance), and the distance between two clusters of observations (agglomeration).

### Distance

A distance metric is a non-negative number which measures the difference between two objects. A value of 0 denotes no difference, with higher values corresponding to larger differences. The most common measure of distance calculates the difference in location, with 0 indicating that the two objects are at the same location. This is known as Euclidean distance, and is the default for all heat map functions.

(1) $$d_{Euclidean}\left(\text{x},\text{y}\right)=\sqrt{{\left(x_{1}-y_{1}\right)}^{2}+...+{\left(x_{n}-y_{n}\right)}^{2}}$$

**Figure 1:**
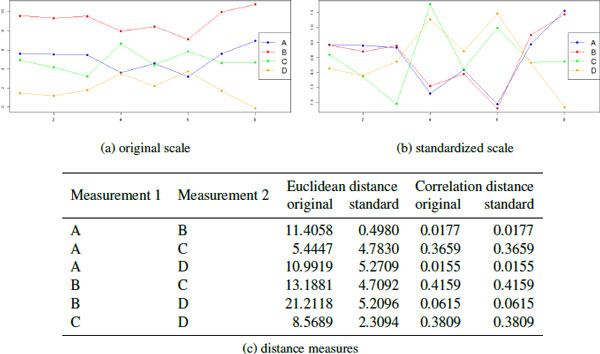
**Distance Measures**. A simulated example of distance measurements using 4 measurements on 8 samples. On the original scale, measurements in A and C are closest in location, while A and B are the most correlated. On the standardized scale, correlation distance does not change, but measurements A and B now are very similar in location. Note that D has a large negative correlation with the A and B, so its correlation distance (using **Equation 2**) is low.

For biological data, the most dominant variation in the data often occur across the features (rows) of the data matrix. Normally, these differences are not interesting, especially in LC-MS/MS data where the intensity of a protein or peptide may be due to many different causes. Rather, it is the changes in protein (or peptide) concentration across the spectra that is of interest. Using Euclidean distance, this variability can cause features with similar profiles to be treated as more distant than those with different profiles with similar mean intensities (Figure 1). There are two strategies used to address this issue.

One solution is to use a distance metric based on the correlation between profiles instead of change in location. Correlation measures the degree to which two variables increase and decrease together, with a range of [-1,1]. More extreme values demonstrate a strong relationship and values close to 0 indicate a weaker (or non-existent) relationship. The sign indicates whether the two variables increase together (positive) or one increases when the other decreases (negative). However, by default this is not a distance metric because it includes negative values, and increasingly similar patterns are represented by values further from zero instead of closer to 0. Two different methods are used to convert correlations to correlation distances.

(2) $$d_{cor}\left(\text{x},\text{y}\right)=1\;-|\text{cor}(\text{x},\text{y})|$$

(3) $$d_{cor}\left(\text{x},\text{y}\right)=1\;-\text{ cor}\left(\text{x},\text{y}\right)$$

Using **Equation 2**, if two variables have a strong relationship, they have a closer distance, regardless of whether they are both up-regulated together, or if one is up regulated when the other is down-regulated. In **Equation 3**, two variables must have a strong positive relationship to have a close distance: they must both be up-regulated together. Both definitions are useful in proteomic data sets where the actual measurements are not important, but the change in measurement from spectra to spectra is. Correlation distance captures whether the profile of up-regulation and down-regulation across spectra is the same for two proteins.

The second strategy for handling the variability across features is to standardize each row (feature) so that it has a mean of 0 and a standard deviation of 1. This removes systematic differences between different features with the same profile, so that proteins with the same profile have a small Euclidean distance. This strategy is illustrated by Figure 1(b). Standardizing the scale does not affect the correlation distance (Figure 1(c)).

The default distance function used by all heat map implementations is Euclidean distance, and can be modified using the distfun parameter. To change the distance function, a stand-alone (single parameter) distance function is required, such as those included in the bioDist package [9], which is a part of Bioconductor [8]. Alternatively, more flexibility can be gained by defining the distance function manually. For example, the cor.dist function in the bioDist package provides a measure of correlation distance, but cannot handle any missing data. Manually defining correlation distance allows more flexibility in how missing data is handled.

cor.dist <- function(x){

   as.dist(1- abs(cor(t(x),

      use="pairwise.complete.obs")))

}

### Agglomeration

Agglomeration is the process by which clusters are merged into larger clusters: and more importantly, determining which clusters should be merged. Unfortunately, measuring the distance between clusters is more complicated than measuring the distance between objects. Agglomeration methods must be compatible with the distance metric, because it is possible to merge two objects, two clusters, or a cluster and an object at most stages in the algorithm. It also must produce consistent results: the height of a cluster should never be smaller than the heights of the two clusters which were merged to create it. Several algorithms have been developed which meet these properties, of which two are especially common.

**Figure 2:**
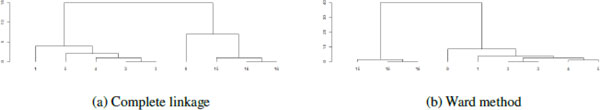
**Agglomeration Methods**. An illustration of the difference between (**a**) complete linkage and (**b**) the Ward method of agglomeration. When merging 3 groups into 2 using complete linkage, 16 - 9 = 7 > 9 - 1 = 8, so 9 is grouped with the larger numbers. Using the Ward method, the groups {1, 3, 3, 4, 5}, {9, 15, 16, 16} produces an *ESS*^2 ^= 42.8, while {1, 3, 3, 4, 5, 9}, {15, 16, 16} produces *ESS*^2 ^= 37.5, so the second merge strategy is used.

The default metric used by the heat map function is called complete linkage. For two clusters, X and Y, it is calculated as

(4) $$D_{complete}\left(\text{X},\text{Y}\right)=\text{max}\left(d\left({\text{x}}_{\text{i}},{\text{y}}_{\text{j}}\right)\right)$$

for all *x_i _*in X and *y_j _*in Y. In words, the distance between two clusters is calculated as the distance between the two most distant points in each cluster (Figure 2(a)). This type of agglomeration method was developed from networking models, where the distance between two nodes is measured by the longest path between them. Variations on this method use the minimum or average distance between all pairs.

The Ward method [10] has a more statistical basis (Figure 2(b)). Using this metric, the distance between groups is defined as the amount of information lost (or error created) by summarizing the objects into *n *clusters. At each step, the merge is chosen which minimizes this information loss, which is defined by the error sum of Squares (*ESS*).

(5) $$\text{min}\;ESS,ESS=\overset{n}{\underset{j=1}{\sum }}\left(\overset{n_{j}}{\underset{i=1}{\sum }}x_{i}^{2}-\frac{1}{n_{j}}{\left(\overset{n_{j}}{\underset{i=1}{\sum }}x\right)}^{2}\right)$$

where *j *is an index for each cluster, *n_j _*is the number of objects in cluster *j*, and *i *is an index for each object in cluster *j*.

As an example, consider Figure 2b. After the 6th step, there are 3 clusters:

(6) {1,3,3,4,5},{9},{15,16,16}

There are three ways to take these 3 clusters and merge them into 2

(7) {1,3,3,4,5},{9,15,16,16}

(8) {1,3,3,4,5,9,},{15,16,16}

(9) {9},{1,3,3,4,5,15,16,16}

Calculating the *ESS *for each set of groups, we see

(10) $$ESS_{\left\{1,3,3,4,5\right\},\left\{9,15,16,16\right\}}=42.8$$

(11) $$ESS_{\left\{1,3,3,4,5,9\right\},\left\{15,16,16\right\}}=37.5$$

(12) $$ESS_{\left\{9\right\},\;\left\{1,3,3,4,5,15,16,16\right\}}=300.875$$

The second merge produces a smaller *ESS*, and so that is the clustering used. In general, the Ward method tends to produce tight concentric clusters.

The purpose of reordering the data is to cluster rows and columns with similar profiles so that patterns among the features and spectra can be easily observed. The most important consideration in this process is ensuring that the distances efficiently measure the similarity across spectra in biologically meaningful ways, i.e. without being influenced by systematic differences in features caused by technical aspects of detection via mass spectrometry. This can be accomplished by standardizing the data or using correlation distance. A good agglomeration method will cause patterns to be easily discerned across features and spectra. The same method may not be ideal for all data sets, so it is important to explore several to see what works best. All heat map functions default to using the hclust function to perform agglomeration. Within the hclust function, the agglomeration method is specified using the method option, but this cannot be adjusted it is called through the heat map. The simplest way to adjust this is to create a wrapper for the hclust function (or any other preferred agglomeration algorithm) with the desired method. For example, a wrapper which calls hclust using the Ward method would be written as:

hclust.ward <- function(x) {

  hclust(x,method="ward")

}

This function is specified using the hclustfun option in all heat map implementations.

## Image representation

**Figure 3:**
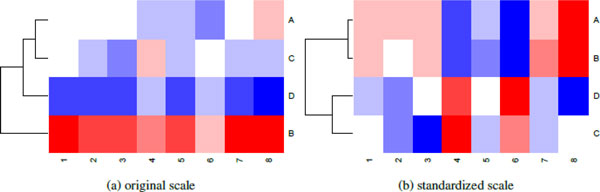
**Color mapping**. Heat maps produced using the simulated data from Figure 1 and Euclidean distance. In **(a)**, the colors are mapped to the original data, in **(b) **the colors are mapped to row-scaled data. Using row-scaled data, it is much easier to see that the patterns in A and B are the same. Using the original data, the differences in intensity between each row dominate the image. By default, data is row-scaled.

Image representation is the process of mapping the intensity range of the data to a color palette. A mapping will assign a specific range of values to a particular color, for example suppose we map all numbers in the range (5,8) to green. Mappings are constant across the entire data set: any value between 5 and 8 in all columns and all rows of the data matrix are mapped to green. Similar to the problem with distance calculations, a mapping which uses the original data is likely to be dominated by differences in the range of each feature. Figure 3 shows this for the simulated data presented in Figure 1. In Figure 3(a), the color scale is mapped to the original data while in Figure 3(b), the data has been scaled across rows before mapping to colors. Using the original data, the most dominant characteristic of the heat map is the difference in intensity across features. Using the standardized data, these differences are removed so that it is easy to see that rows **A **and **B **have a similar pattern across samples. Unless the actual numerical values in the data matrix have an explicit meaning, row scaling is usually advisable, and the heat map functions typically do this by default.

### Color mapping

Once any scaling has been performed, color mapping assigns breaks to the data range. Breaks are the transition points between one color in the palette and the next. By default, the data range is dividing into *n *equally spaced bins, requiring *n *+ 1 breaks (including the minimum, *n *- 1 internal breaks, and the maximum). The number *n *varies across the different heat map functions, but is normally between 9 and 20. Equally-spaced breaks are ideal for compact data sets without outliers. In many real data sets, especially proteomics data sets, outliers can make this representation less effective.

**Figure 4:**
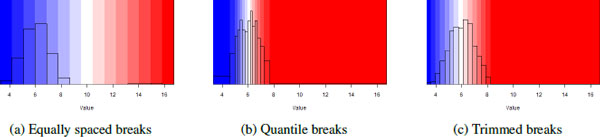
**Breaks**. Breaks are assigned to 1000 randomly generated *N*(6, 1) data points plus 20 randomly generated *N*(15, 1) data points using 15 bins and 3 different strategies. In the default scheme, **(a)**, spaces the breaks evenly across the entire data range. In **(b)**, breaks are chosen to ensure that roughly the same number of data points fall within each break. In **(c) **the top 1% of the data is placed in a single bin while the remainder is placed in equally spaced bins.

In the presence of outliers, equally spaced bins are often inefficient, as seen in Figure 4(a). In this example, 1000 data points were generated from a normal distribution with mean 6 and 20 data points were generated from a normal distribution with mean 15. Using a color scale with 15 equally spaced bins, 5 are completely empty, 4 contain the 20 outliers, and 6 are used to represent the remaining 1000 data points. An alternative strategy is to define bins based on percentiles. Using percentiles, breaks are chosen to ensure that approximately the same number of data points are assigned to each bin (Figure 4(b)). While percentiles ensure that only one bin is used to represent the outliers, it tends to over-correct the problem: in highly populated mid-ranges each bin is likely to cover a very small range. A mixed strategy usually works best: the outliers are placed into one or two bins defined by the top (and bottom) *p^th ^*percentiles, while the remaining bins are equally spaced between these percentiles. This is shown in Figure 4c. The heatmap_2 and heatmap_plus functions in the Heatplus package contain the option trim=p which creates bins for values below the *p^th ^*percentile and above the (1 - *p*)*^th ^*percentile. Using other functions, these breaks must be defined explicitly.

Color mapping is controlled by the breaks option. A vector of *n *+ 1 monotonically increasing numbers is required to map the data set to *n *colors, with the first number no larger than the minimum value in the data set and the last number no smaller than the maximum value in the data set. Breaks are always applied to the final data after any scaling has been performed, so if manually specified breaks are desired, the data matrix must be scaled outside of the heat map function, and the breaks must be calculated using the scaled data. By default, each heat map function uses equally spaced breaks.

### Color palette

**Figure 5:**
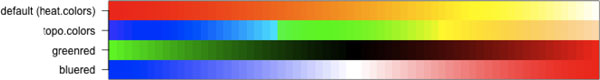
**Color palettes**. A selection of pre-defined color palettes available in R.

The color palette is the set of colors used to represent the values of the data matrix. This is normally chosen to gradually shift from one color representing low values to a second color representing high values, sometime by way of a third color representing intermediate values. In the default scheme, low values are represented by red and high values are represented by yellow using the heat.colors palette. This palette and a few other popular choices are shown in Figure 5.

Several packages in R, including gplots can be used to generate a custom color palettes by designating the low, middle, and high colors. For example, the following code will create a vector of 64 colors from pink to blue, going through brown in the middle:

#Requires gplots package

my.colors <- colorpanel(64,low="pink",

  mid="brown",

  high="blue")

While the choice of color palette is largely personal preference, two considerations are worth mentioning. First, although the green-black-red color scheme is extremely common due to its relation to red/green channels in microarray experiments, it cannot be interpreted by the color blind. For this reason, it should be avoided. Second, dark colors can be harder to distinguish from one another compared to light colors, as evidenced by comparing the bluered and greenred color schemes in Figure 5. In order to maximize the ability to distinguish colors in midranges, using a light "mid" color may be preferable.

The gplots package also contains pre-defined palettes particularly common in high throughput biological experiments including redgreen, greenred bluered, and redblue. This author prefers the bluered scheme, where low values are blue, middle values are white, and high values are red. This palette will be utilized throughout the remainder of this tutorial.

## Extras

Although the basic components discussed above are shared by all heat map functions, the implementation of other features varies significantly. While it is beyond the scope of this tutorial to provide an in-depth review of all the features of all the functions, three features in particular require mentioning.

### Color key

A color key is used to show the map between the data range (after scaling) and the colors. For any matrix, this can be useful in demonstrating which color(s) represent smaller values and which represent larger values. It becomes far more important when the data values have an explicit meaning. For example, a correlation matrix may contain values in the range of -1 and 1. When all the correlations contained in the matrix are greater than 0, using a blue-white-red color scheme without careful consideration of the breaks could produce a misleading visualization. By including a color key, such a mistake can be caught and corrected.

The functions heatmap_2, and heatmap.2 will produce legends as part of the heat map graphics. Color keys can be created manually for functions which will not create them or in cases where a non-standard key may be desired, as shown in Figure 4.

### Group labels

Group labels provide the final piece of information for a heat map. They allow us to incorporate known group memberships (e.g. the disease group or gender for a sample, the protein membership of a peptide, or the annotation of a protein) into the heat map picture. This information can be used to determine (1) whether groups of samples or features with the same group membership tend to cluster together and (2) if subgroups of samples or features with the same group membership have a distinct profile.

Each heat map function has unique methods available for displaying group information, so its useful to demonstrate each function separately. Consider a simulated data set with 24 samples and 10 features. The 24 samples are each associated with two different grouping variables: one which takes on 2 values (e.g. male/female) and one with 3 values (e.g. 3 disease groups). For the purpose of illustration, 5 features are associated with each variable.

**Figure 6:**
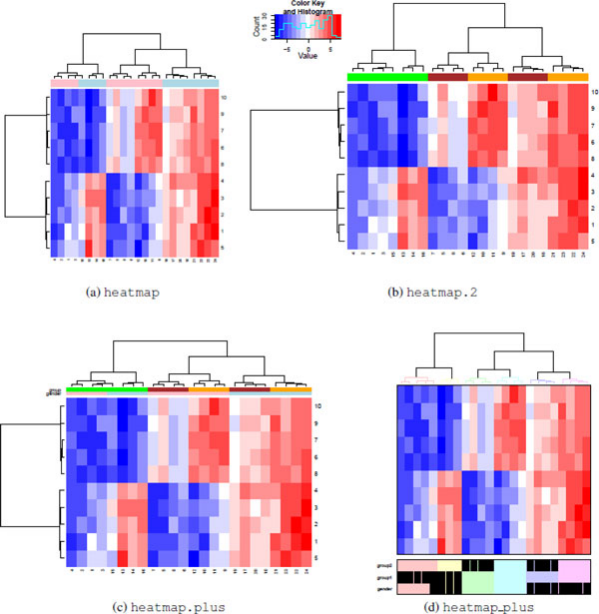
**Group labels**. Rows and columns can be labeled using all heat map functions, but the implementation varies. The heatmap (a) andheatmap.2 (b) functions are limited to displaying a single color bar.The heatmap.plus (c) function can display a matrix of color bars. The heatmap_plus (d) function can display a data frame of binary variables. While heatmap.2 and heatmap.plus can produce a rectangular image, both heatmap and heatmap_plus produce a square image as output.

The heatmap and heatmap.2 functions allow a color vector with group information for columns and rows to be added to the plot using the options ColSideColors and RowSideColors respectively. In Figure 6(a) and 6(b), ColSideColors is used to show how samples are divided by gender **Figure (a) **and group **Figure (b)**. To display more than two groups on the same heat map, the heatmap.plus function uses a data matrix of colors instead of a vector in Figure 6(c). It is possible to show colors on both the rows and columns of the image, but both must be matrices with at least 2 columns and rows equal to the rows (or columns) of the image.

The heatmap_plus function can display a binary (0,1) data frame with group information for each column using the addvar option. Each 1 in the data frame is displayed as a black box under the image plot. The h option is unique to the heatmap_plus function. Clusters of data points are defined by cutting the dendrogram at the specified height, with each cluster shown using a different color in both the dendrogram and in the group display. This is illustrated in Figure 6(d).

### Layout

The image layout determines the amount of space in the graphics window devoted to the dendrograms, group labels, image matrix, color key (if applicable) and margins. The heatmap_2 and heatmap_plus functions, based off of an early version of the heatmap function, provide very little control over the image layout: each component (if available and incorporated) has a fixed size and shape in the graphic, which cannot be modified. For example, a data matrix with dimensions 20 × 20 and a data matrix with dimensions 40 × 500 will both display in the heat map as a square - but the latter data matrix will contain smaller, rectangular boxes to represent each entry. The most control is possible with the heatmap.2 function. In this function, it is possible to explicitly define the fraction of space in the graphic windows for each component of the heat map. By default, heatmap.2 and heatmap.plus, fill the entire graphics window, so by changing the size of the output window or file, each cell can be made approximately square. The heatmap function is less predictable, with the results depending on the operating system.

## Example

To demonstrate the use of heat maps in an analysis, we will use the Prostate2000Peaks data set, which is available in the msProstate package [4] in R. This case study consists of 779 aligned, background-corrected, and normalized peaks detected in 652 spectra using SELDI TOF mass spectrometry [11]. The 652 spectra consist of duplicate measurements on 326 serum samples from 167 subjects with prostate cancer (PCA), 77 subjects with benign prostate hyperplasia (BPH), and 82 normal controls.

## Data preparation

Before any analysis can begin, the data must be formatted appropriately. At the very least, the data must be organized into a *n *× *p *matrix where *n *is the number of spectral features and *p *is the number of samples. This is often insufficient to produce a good heat map, particularly when the data has a non-symmetric distribution and missing values - both common situations when working with proteomic data. The Prostate2000Peaks data set is typical example of this, with a log normal distribution and a large number of zeros assumed to come from unobserved peaks.

Excluding the zeros, the range of data in the Prostate2000Peaks data set is (0.006, 79.234), which translates to a range of (-7.381, 6.308) on a log_2 _scale. Since log_2_(0) = -∞, the zeros must be removed or replaced with a different value before applying the log transformation. Because the data reordering and color mapping steps are performed independently, different strategies can be used for each component of the heat map. Initially, we replace each zero with an NA, which designates missing data in R.

The distance function determines how robust the heat map function is to missing data. At a minimum, it is necessary to have at least one observed value in common for two samples or features to calculate a distance. In the Prostate2000Peaks data set, we address this by filtering out all features in which over 90% of the data is missing or fewer than 4 observations are found for any of the three disease groups. For the remaining 139 spectra, we replace the missing data with a low value for the purpose of calculating distances.

## Example 1: Simultaneous clustering of samples and features

In the Prostate2000Peaks data set, the primary goal of clustering spectra is to determine whether there is a characteristic abundance pattern in each type of spectra. The choice of distance function and agglomeration method should be chosen to aid in this visualization, and multiple methods can be compared. Only one group label is available (type of spectra), so the functions heatmap or heatmap.2 provide sufficient group information. In this case, the two advantages of using heatmap.2 is that it provides an option to change the cell color of missing values, and generates a color key.

While entire books have been written on the subject of missing data (e.g., [12]), the large quantities of missing data often encountered in mass spectrometry data make it necessary to briefly address the issue here. Several characteristics affect how missing data is incorporated into the heat map. In mass spectrometry experiments, missing data is typically assumed to result from unobserved or unquantifiable peaks. Consequently, the observed data and the missing data have different properties: missing data would have had lower values if it had been observed. In a heat map, missing data affects how the rows are standardized, the calculation of distances between rows (and columns), and how the matrix entries are represented in the color scheme.

Missing data affects row standardization because the center and standard deviation of the data are determined by the observed data. When the smallest values in the row are unobserved, these estimates are biased. In particular, the calculated standard deviation is likely to underestimate the true standard deviation, which magnifies differences in the observed data (after standardization). Alternatively, replacing the missing data with a low value can have a noticeable impact on the resulting heat map. Choosing a replacement value without understanding how the data was quantified will lead to bias. In this data set, the data is standardized while treating unobserved values as missing.

To measure the distance between two objects, the two objects must have at least 1 measurement in common. So to measure the distance between two samples, there must be one feature in which both samples have non missing data, and two features must have one sample on which both are measured. In this particular data set, this restriction would greatly reduce the number of features we could display. Since this would greatly diminish the value of the heat map, we know that the unobserved values are low, and we are primarily interested in visualization (and not a statistical test), we can justify the decision to impute the missing data to calculate the distance between rows. The missing values are replaced with a value of -10, which corresponds to approximately 0.001 on the original scale (and much lower than the minimum observed value of 0.006). Both dendrograms are created independently of the heat map using correlation distance and the Ward method of agglomeration. Creating them independently allows us use the data matrix with missing values in the call to heatmap.2 so that we can use the options heatmap.2 offers for displaying missing data.

**Figure 7:**
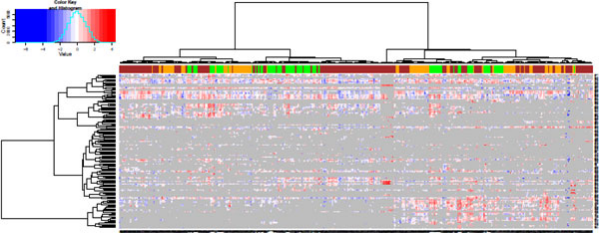
**Features and samples in the ****Prostate2000Peaks****data set**. The spectral features (rows) and samples (columns) from the Prostate2000Peaks data set. Columns are colored according to group (green = control, orange = BPH, brown = PCA). In the heat map image, gray indicates a missing value. The dendrograms are created using correlation-based distances and the Ward method of hierarical clustering.

For this heat map, gray is chosen to represent missing values. The breaks are specified to group the top and bottom 0.2% of the data into separate bins, with the rest of the data placed into equally spaced bins. The final heat map is shown in Figure 7, with a larger version available in the supplementary materials.

### Interpretation

We focus on interpreting the behavior of the samples in Figure 7, shown in columns. While it is clear that there are rows with similar profiles, the absence of any additional data on the spectrum make it difficult to analyze the features further. The colors at the top of the heat map show the disease group (green = control, orange = BPH, brown = PCA). It is immediately obvious that the primary divisions between these samples is not the disease group. However, samples from the same disease group do tend to form small clusters with other samples from the same disease group. Furthermore, it is apparent that some of the BPH and PCA samples form clusters which exclude the healthy controls. Focusing on understanding the difference between these samples and those that cluster with healthy controls may provide insight which can be explored further through follow-up experiments.

## Example 2: Presenting significance results

Most statistical analyses involve one or more tests of statistical significance. In a mass spectrometry data set, the same tests is usually performed separately for each feature, or on the group of spectrum coming from the same protein. When multiple tests are performed on each protein or feature, a heat map can be used to organize and display the results. For the Prostate2000Peaks data set, we use t-tests to compare the disease groups in a pairwise fashion, with 3 comparisons total: PCA - BPH, PCA - control, and BPH - control. Each t-test is performed separately for each feature, resulting in two results matrices: one with the *p*-values, and a second with the *t*-statistics.

**Figure 8:**
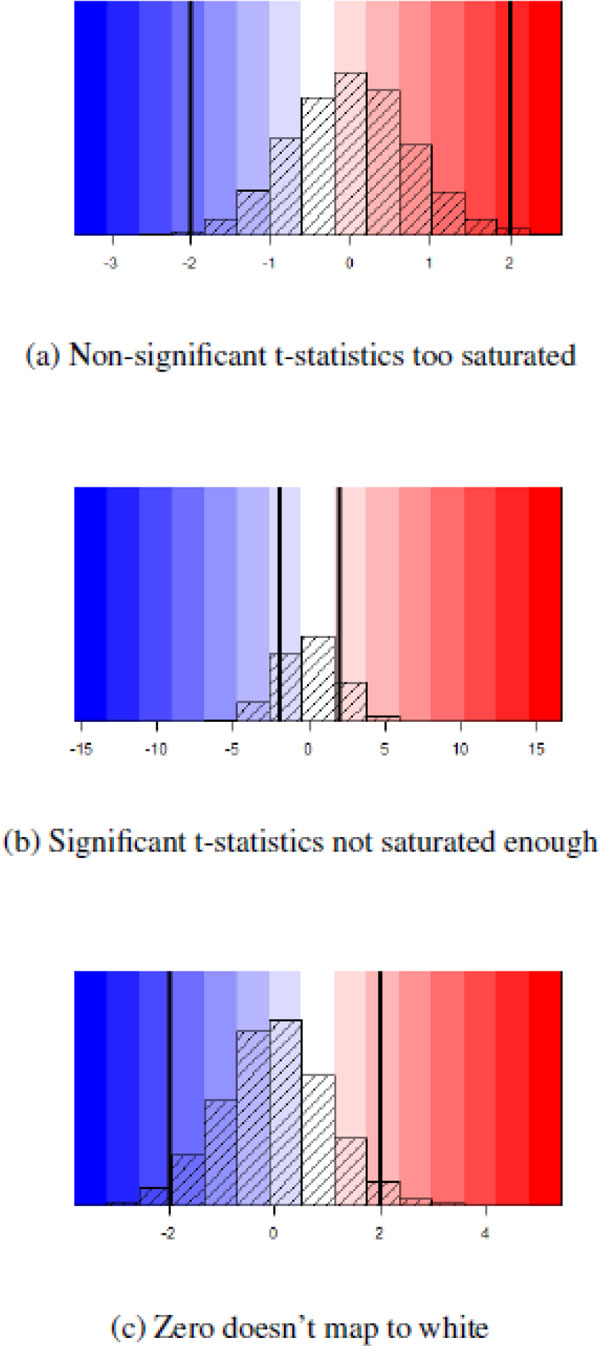
**Pitfalls in using *t*-statistic based breaks**. Three possible problems when deriving a color scale based on *t*-statistics. The veritcal lines mark (-2, +2), outside of which we will consider the *t*-statistics significant. In **(a)**, marginally significant *t*-statistics are shown in extremely saturated colors, because the range of *t*-statistics does not extend far past the significance threshold. In **(b)**, a small number of *t*-statistics extends far past the significance threshold, resulting in less significant *t*-statistics to appear extremely light (e.g. the colors assigned to +5 and -5). In **(c)**, the range of *t*-statistics is not symmetric around 0, resulting in slightly positive *t*-statistics (approximately 0 - 1) to have a light blue color instead of white or light red.

The most important component of presenting significance results is the map between the matrices and the color palette. Specifically, a 3-color palette (e.g. the blue-white-red palette used here) should have a natural interpretation: white should indicate non-significance, while progressively more saturated values of blue and red should indicate increased significance, with the color dependent upon the sign of the *t*-statistic. This will prevent three possible interpretation pitfalls which can occur if the heat map is created using *t*-statistics with equally spaced breaks, as shown in Figure 8. Finally, when the number of degress of freedom is different for each *t*-statistic, as is the case when the number of samples in each comparison varies, the significance cut-off may be different for each comparison. For all of these reasons, displaying raw *t*-statistics in a heat map is not recommended. Instead, compute

(13) $$r=-{\text{log}}_{10}\left(p\right)\times \text{sgn}\;\left(t\right)$$

that is, taking the log of the *p*-value, and multiplying it by the sign of the *t *statistic. The first transformation ensures that large *p *values (close to 1) are mapped near 0 while smaller *p *values are mapped to larger numbers. The second transformation ensures that the sign of the new matrix is the same as the sign of the *t*-statistic. Working with *p*-values has one additional benefit: multiple comparison correction is easy to incorporate.

Typically, a *p*-value is not considered significant unless it is below a specific threshold, where 0.1, 0.05, and 0.01 are typical starting points. Using this as a starting point, we consider the following breaks, based on significance:

(14) (0.000001,0.00001,0.0001,0.001,0.01,0.05,0.1,1)

**Figure 9:**
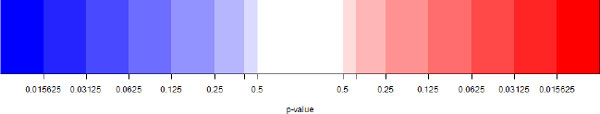
***p*-value based breaks**. A color-scale based on *p*-value-based cut-offs. Increasingly more saturated hues are associated with lower *p*-values. The color is based on the sign of the *t*-statistic.

This color scale is independent of the distribution of the *p*-values. This prevents the pitfalls discussed earlier, and ensures that we have a standard basis for understanding any heat map presentation of results. The resulting color scale is shown in Figure 9.

Creating this type of heat map requires the following procedure:

1. Calculate the *t*-statistics and *p*-values, and arrange as a matrix. Perform multiple comparison correction on the *p*-values.

2. Calculate the matrix of displayed values using Eq.13.

3. Calculate the inner breaks by applying Eq.13 to the p-values above.

4. Calculate the minimum break as the minimum display value or -7, whichever is smaller. Calculate the maximum break as the maximum display value or 8, whichever is larger. Put all of these values into a single monotonically increasing breaks vector.

5. Create the heat map. Note that the option scale="none" must be used. In the Prostate2000Peaks data set, the trace option is used to emphasize how significant some results are without affecting the color scale.

### Interpretation

**Figure 10:**
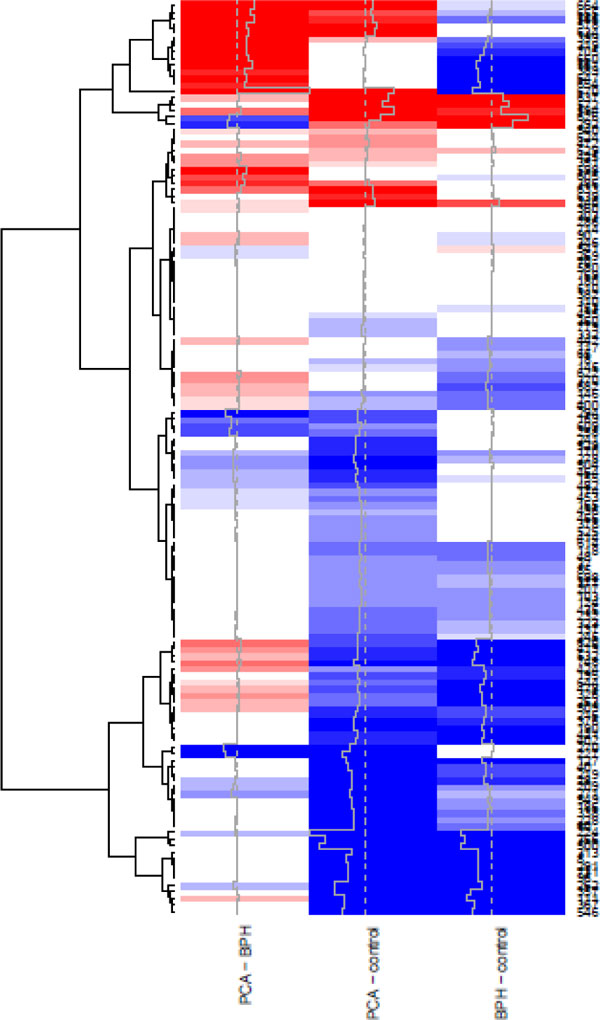
**Significance results for the ****Prostate2000Peaks****data set**. A heat map is used to display the significance results when performing pairwise comparisons between the disease groups, using the color key in Figure 9.

We see the significance results for the Prostate2000Peaks data set in Figure 10. The option trace in the heatmap.2 function produces the gray line, which provides an alternative means of showing the results matrix. From the picture, we see that we can find significant differences between all three disease groups. The bottom cluster of features have significantly lower abundances in disease (PCA and BPH) when compared to healthy subjects, thus presenting a disease signature. At the very top, we can see a second group of features which are significantly higher in subjects with PCA when compared to controls, and another group which are significantly lower in subjects with BPH when compared to controls. These two groups of features appear to differentiate the three groups. These groups of spectra form a starting point for further experiments and analyses into the proteins which differentiate these three disease groups.

## Competing interests

The author declares no competing interests.

## Supplementary Material

Additional File 1 The file contains all the source code necessary to reproduce the figures in this tutorial. Click here for file

Additional file 2 **The file summarizes features available in each heat map function**. Click here for file

Additional file 3 A larger version of Figure 7. Click here for file
