# Supplementary material for: A tutorial in displaying mass spectrometry-based proteomic data using heat maps
Source: BMC Bioinformatics. 2012 Nov 5;13(Suppl 16):S10. doi: 10.1186/1471-2105-13-S16-S10 (PMC3489527; doi:10.1186/1471-2105-13-S16-S10)
Supplement: Additional File 1 — The file contains all the source code necessary to reproduce the figures in this tutorial. [file 1471-2105-13-S16-S10-S1.pdf]

```
#####################################
# ADDITIONAL FILE 1: R SCRIPTS
#####################################



# set working directory
setwd("D:/bmc_heatmap")


#####################################
# Figures 1 and 3
#####################################

n <- 8
samples <- 1:n

y1 <- rnorm(n,mean=5,sd=1.5)
y2 <- y1 + rnorm(n,mean=4,sd=.2)
y3 <- rnorm(n,mean=5,sd=1.5)
y4 <- rnorm(n,mean=7,sd=0.3) - y1

r <- range(c(y1,y2,y3,y4))

png("images/figure1a.png",
  width=480*2)
plot(y1 ~ samples,type="b",col="blue",pch=20,ylim=r,xlab="",ylab="",xlim=c(1,8.75),cex=2)
lines(y2 ~ samples,type="b",col="red",pch=20,cex=2)
lines(y3 ~ samples,type="b",col="green",pch=20,cex=2)
lines(y4 ~ samples,type="b",col="orange",pch=20,cex=2)
legend("right", c("A","B","C","D"),col=c("blue","red","green","orange"),lty=1,pch=20,cex=2)
dev.off()

z1 <- scale(y1)
z2 <- scale(y2)
z3 <- scale(y3)
z4 <- scale(y4)

rz <- range(c(z1,z2,z3,z4))

png("images/figure1b.png",
  width=480*2)
plot(z1 ~ samples,type="b",col="blue",pch=20,ylim=rz,xlab="",ylab="",xlim=c(1,8.75),cex=2)
lines(z2 ~ samples,type="b",col="red",pch=20,cex=2)
lines(z3 ~ samples,type="b",col="green",pch=20,cex=2)
lines(z4 ~ samples,type="b",col="orange",pch=20,cex=2)
legend("right", c("A","B","C","D"),col=c("blue","red","green","orange"),lty=1,pch=20,cex=2)
dev.off()

round(cor.dist(rbind(y1,y2,y3,y4)),4)
round(dist(rbind(y1,y2,y3,y4)),4)
round(dist(t(cbind(z1,z2,z3,z4))),4)
round(cor.dist(t(cbind(z1,z2,z3,z4))),4)

data.mat <- rbind(y1,y2,y3,y4)
rownames(data.mat) <- c("A","B","C","D")

data.std <- t(cbind(z1,z2,z3,z4))
rownames(data.std) <- c("A","B","C","D")

pdf("images/figure3a.pdf",
  width=5,
  height=3
)
heatmap.2(data.mat,
  Colv=FALSE,
  col=bluered(9),
  scale="none",
  trace="none",
```

```
  key=FALSE,
  cexRow=1,
  cexCol=1,
  lwid=c(1,5),
  lhei=c(1,15),
  margins=c(2,2),
)
dev.off()

pdf("images/figure3b.pdf",
  width=5,
  height=3
)
heatmap.2(data.std,
  Colv=FALSE,
  col=bluered(9),
  scale="none",
  trace="none",
  key=FALSE,
  cexRow=1,
  cexCol=1,
  lwid=c(1,5),
  lhei=c(1,15),
  margins=c(2,2),
)
dev.off()


######################################
# Figure 2
######################################

ward.example <- c(1,3,3,4,5,9,15,16,16)
names(ward.example) <- ward.example

dend.ward <- as.dendrogram(hclust(dist(ward.example),method="ward"))
dend.complete <- as.dendrogram(hclust(dist(ward.example)))


ward.o <- order.dendrogram(dend.ward)
complete.o <- order.dendrogram(dend.complete)


png("images/figure2a.png",
  width=480*2,height=480/1.5)

plot(dend.complete,leaflab="none",xlab="")
axis(1,at=1:length(ward.example),labels=ward.example[complete.o],lty=0,pos=0)
dev.off()

png("images/figure2b.png",
  width=480*2,height=480/1.5)
plot(dend.ward,leaflab="none",xlab="")
axis(1,at=1:length(ward.example),labels=ward.example[ward.o],lty=0,pos=0)

dev.off()

g1 <- c(9,15,16,16)
g2 <- c(1,3,3,4,5,9)

ess <- function(x){sum(x^2) - sum(x)^2 / length(x)}

ess1 <- ess(g1) + ess(g2[-length(g2)])
ess2 <- ess(g1[-1]) + ess(g2)



######################################
# Figure 4
```

```
######################################

x <- rnorm(1000,mean=6)
x1 <- c(x,rnorm(20,mean=15))
b1 <- seq(min(x1),max(x1),length=16)

b2 <- quantile(x1,probs=seq(0,1,length=16))

q1 <- quantile(x,probs=0.99)
b3 <- c(seq(min(x1),q1,length=15),max(x1))
color.palette <- bluered(15)

png("images/figure4a.png",height=480/1.5)
    image(x=b1, y=c(0,.5), z=matrix(1:length(color.palette),nrow=length(color.palette)),
         ylab="", xlab="Value", yaxt="n", col=color.palette)
    hist(x1, breaks=b1, add=TRUE,freq=FALSE)
dev.off()

png("images/figure4b.png",height=480/1.5)
    image(x=b2, y=c(0,.5), z=matrix(1:length(color.palette),nrow=length(color.palette)),
         ylab="", xlab="Value", yaxt="n", col=color.palette)
    hist(x1, breaks=b2, add=TRUE,freq=FALSE)
dev.off()

png("images/figure4c.png",height=480/1.5)
    image(x=b3, y=c(0,.5), z=matrix(1:length(color.palette),nrow=length(color.palette)),
         ylab="", xlab="Value", yaxt="n", col=color.palette)
    hist(x1, breaks=b3, add=TRUE,freq=FALSE)
dev.off()

######################################
# Figure 5
######################################

showpanel <- function(col.pal,n,...)
{
  image(z=matrix(1:length(col.pal), ncol=n), col=col.pal, xaxt="n", yaxt="n",...)
}

brewer <- brewer.pal(9,"YlOrRd")

tmp <- c(
    bluered(64),
    greenred(64),
    topo.colors(64),
    heat.colors(64)
)
png("images/figure5.png",
    height=480 * .3,
    width=480 * 1.5)
par(mar=c(2,10,2,2))
showpanel(tmp,4)
axis(2,
    at=0:3/3,
    labels=c("bluered",
        "greenred",
        "topo.colors",
        "default (heat.colors)"),
    las=2)
dev.off()

######################################
# Figure 6
######################################

# Simulated data matrix
gender.base <- rep(c(-3,3),each=12) + rnorm(24,sd=1.5)
gender.obs <- t(matrix(gender.base + rnorm(24*5,sd=1),ncol=5))
```

```r
group.base <- rep(c(-5,0,5),each=4,length=24) + rnorm(24,sd=1.2)
group.obs <-  t(matrix(group.base + rnorm(24*5,sd=.75),ncol=5))

data.matrix <- rbind(gender.obs,group.obs)

# Column Side Colors
# Individual vectors
gender <- rep(c("pink","lightblue"),each=12)
group <- rep(c("green","brown","orange"),
    each=4,length=24)
# Matrix for heatmap.plus
color.matrix <- cbind(gender,group)
# Data frame for heatmap_plus
color.df <- data.frame(
  gender= gender== "lightblue",
  group1=group == "green",
  group2=group == "brown"
)


gender.base <- rep(c(-3,3),each=12) + rnorm(24,sd=1.5)
gender.obs <- t(matrix(gender.base + rnorm(24*5,sd=1),ncol=5))

group.base <- rep(c(-5,0,5),each=4,length=24) + rnorm(24,sd=1.2)
group.obs <-  t(matrix(group.base + rnorm(24*5,sd=.75),ncol=5))

data.matrix <- rbind(gender.obs,group.obs)

pdf("images/figure6a.pdf")
heatmap(data.matrix,
    hclustfun=hclust.ward,
    col=bluered(15),
    ColSideColors=gender,
# ColSideColors=group,
)
dev.off()


pdf("images/figure6b.pdf",
  height=6,
  width=8)
heatmap.2(data.matrix,
    hclustfun=hclust.ward,
    col=bluered(15),
#   ColSideColors=gender,
 ColSideColors=group,
    trace="none" # specific to heatmap.2
)
dev.off()

pdf("images/figure6c.pdf",
  height=6,
  width=8)
heatmap.plus(data.matrix,
    hclustfun=hclust.ward,
    col=bluered(15),
    ColSideColors=color.matrix,
)
dev.off()

pdf("images/figure6d.pdf")
heatmap_plus(
  data.matrix,
  hclustfun=hclust.ward,
  addvar=color.df,
  col=bluered(15),
  h=20,
)
dev.off()
```

```
#####################################
# Figure 8
#####################################

# Significance Color scheme fallacies
case1 <- rnorm(10000,sd=.75)
case2 <- c(rnorm(9900,sd=2),rnorm(50,mean=12,sd=2),rnorm(50,mean=-12,sd=2))
case3 <- c(rnorm(9950),rnorm(50,mean=3))

png("images/figure8a.png",height=480/1.5)
case1.breaks <- seq(min(case1),max(case1),length.out=length(display.breaks))
                image(x=case1.breaks, y=c(0,.75), z=matrix(1:n.cols,nrow=n.cols),
                     xlab="",ylab="", yaxt="n", col=bluered(n.cols))
hist(case1,breaks=case1.breaks,add=TRUE,freq=FALSE,density=10)
abline(v=c(-2,2),lwd=3)
dev.off()

png("images/figure8b.png",height=480/1.5)
case2.breaks <- seq(min(case2),max(case2),length.out=length(display.breaks))
                image(x=case2.breaks, y=c(0,.5), z=matrix(1:n.cols,nrow=n.cols),
                     xlab="",ylab="", yaxt="n", col=bluered(n.cols))
hist(case2,breaks=case2.breaks,add=TRUE,freq=FALSE,density=10)
abline(v=c(-2,2),lwd=3)

dev.off()

png("images/figure8c.png",height=480/1.5)
case3.breaks <- seq(min(case3),max(case3),length.out=length(display.breaks))
                image(x=case3.breaks, y=c(0,.5), z=matrix(1:n.cols,nrow=n.cols),
                     xlab="",ylab="", yaxt="n", col=bluered(n.cols))
hist(case3,breaks=case3.breaks,add=TRUE,freq=FALSE,density=10)
abline(v=c(-2,2),lwd=3)
dev.off()


#####################################
# Figure 9
#####################################

# p-value based Color Scale
png("images/figure9.png",width=480*2,height=480/2)
        scale.breaks <- c(all.breaks[1]-1,all.breaks,all.breaks[14] + 1)
                image(x=scale.breaks, y=c(0,1), z=matrix(1:n.cols,nrow=n.cols),
                     xlab="p-value",ylab="", yaxt="n",xaxt="n", col=bluered(n.cols))
                axis(1,all.breaks,2^(-abs(all.breaks)))
dev.off()


#############################################################
# EXAMPLE USING msProstae DATA SET
#############################################################



# Initial steps to create data sets
library(msProstate)
data(Prostate2000Peaks)

# Log transformation of non-zero data
prostate.log <- ifelse(Prostate2000Peaks$intensity <= 0,NA,
    log2(Prostate2000Peaks$intensity))

KEEP <- apply(prostate.log,1,
  function(x){
    obs.pca <- sum(!is.na(x[Prostate2000Peaks$type == "pca" & !no.peaks]))
  obs.bph <- sum(!is.na(x[Prostate2000Peaks$type == "bph" & !no.peaks]))
    obs.ctrl <- sum(!is.na(x[Prostate2000Peaks$type == "control" & !no.peaks]))
```

```
    min(obs.pca,obs.bph,obs.ctrl) >= 4 & (obs.pca + obs.bph + obs.ctrl) > (.1 * ncol(prostate.log))
  }
)
prostate.data <- prostate.log[KEEP,]

# Remove sample for which no peaks were found
no.peaks <- apply(prostate.data,2,function(x)sum(is.na(x))) == nrow(prostate.data)
prostate.data <- prostate.data[,!no.peaks]
column.colors <- c("orange","green","brown")[Prostate2000Peaks$type[!no.peaks]]


##############################################################
# Example 1
##############################################################

# Record location of each NA in the data matrix
prostate.na <- is.na(prostate.data)

# Scale the data
prostate.scale <- t(apply(prostate.data,1,scale))

# Substitude NAs with a suitable value.
# Create a 2nd version of the data matrix NAs replaced with a suitable value (-8)
prostate.scale.na <- prostate.scale
prostate.scale[prostate.na] <- -10

# Create dendrogram using the data without NAs
feature.dend <- as.dendrogram(hclust(cor.dist(prostate.scale),method="ward"))
sample.dend <- as.dendrogram(hclust(cor.dist(t(prostate.scale)),method="ward"))

# Set up breaks
my.breaks <- c(min(prostate.scale.na,na.rm=TRUE),

 seq(quantile(prostate.scale.na,na.rm=TRUE,probs=0.001),quantile(prostate.scale.na,probs=0.999,na.rm=TRUE),length
 .out = 24),
        max(prostate.scale.na,na.rm=TRUE)
)

######################################
# Figure 7
######################################

# Create heat map
pdf("images/figure7.pdf",
  width=20,
  height=8)
    heatmap.2(
        prostate.scale.na,
    Colv=sample.dend,
    Rowv=feature.dend,
        breaks=my.breaks,
        col=bluered(25),
        scale="none",
        ColSideColors=column.colors,
    na.color = 'gray',
      distfun=cor.dist,
        hclustfun=hclust.ward,
        lwid=c(2,8),
        trace="none"
    )
dev.off()

##############################################################
# Example 2
##############################################################

# Define function to compute the t-tests
make.t.test.matrix <- function(x,group1,group2){
  tmp <- t.test(x[group1],x[group2])
```

```
  as.matrix(data.frame(
    statistic=tmp$statistic,
    p.value=tmp$p.value,
    mean.group1=tmp$estimate[1],
    mean.group2=tmp$estimate[2]
  ))
}

# Calculate t-tests between groups

# PCA and PBH
pca.bph <- t(apply(prostate.data,1,
  make.t.test.matrix,
  group1=Prostate2000Peaks$type == "pca" & !no.peaks,
  group2=Prostate2000Peaks$type == "bph" & !no.peaks
))

# PCA and controls
pca.control <- t(apply(prostate.data,1,
  make.t.test.matrix,
  group1=Prostate2000Peaks$type == "pca" & !no.peaks,
  group2=Prostate2000Peaks$type == "control" & !no.peaks
))

# BPH and controls
bph.control <- t(apply(prostate.data,1,
  make.t.test.matrix,
  group1=Prostate2000Peaks$type == "bph" & !no.peaks,
  group2=Prostate2000Peaks$type == "control" & !no.peaks
))


# Create matrices of t-statistics and p-values
t.stats <- cbind(pca.bph[,1],pca.control[,1],bph.control[,1])
p.values <- cbind(pca.bph[,2],pca.control[,2],bph.control[,2])

# Perform multiple comparison correction
p.BH <- apply(p.values,2,p.adjust,method="BH")

# Transform the p-values to display-values
display.data <- -log10(p.BH) * sign(t.stats)

# Label columns
colnames(display.data) <- c("PCA - BPH","PCA - control","BPH - control")

# Calculate break points
p.breaks <- -log10(c(0.1,0.05,10^(-(2:6))))
all.breaks <- c(-p.breaks[length(p.breaks):1], p.breaks)

display.breaks <-c(min(p.breaks - 1,min(display.data)),
  all.breaks,
  max(p.breaks + 1,max(display.data)))

######################################
# Figure 10
######################################

# Create heat map
n.cols <- length(display.breaks) - 1
pdf("images/figure10.pdf",
  height=8,
  width=5)
heatmap.2(
    display.data,
    Colv=FALSE,
    scale="none",
    breaks=display.breaks,
    col=bluered(n.cols),
    cexCol=0.8, # adjusts x-axis font size
```

```
    hclustfun=hclust.ward,
#   trace="none",
  tracecol="gray65",
    key=FALSE,
    lhei=c(1,15)
)
dev.off()
```
