# Supplementary material for: A tutorial in displaying mass spectrometry-based proteomic data using heat maps
Source: BMC Bioinformatics. 2012 Nov 5;13(Suppl 16):S10. doi: 10.1186/1471-2105-13-S16-S10 (PMC3489527; doi:10.1186/1471-2105-13-S16-S10)
Supplement: Additional file 2 — The file summarizes features available in each heat map function. [file 1471-2105-13-S16-S10-S2.pdf]

Feature	heatmap	heatmap.2	heatmap.plus	heatmap_2	heatmap_plus
Data Reordering	×	×	×	×	×
Color dendrograms				×	
Image Representation	×	×	×	×	×
Block separation		×			
Cell labeling		×			
Level trace		×			
Color key/legend		×		×	×
includes histogram		×			
Labeling and grouping	×	×	×	×	×
adjust margins	×	×	×	×	×
label rows/columns	×	×	×		
add group information	×	×	×		×
add multivariate group information			×		×
Add covariate					×
Layout adjustments		×			

Table 1: Image customization includes the ability to incorporate functions such as `abline` to a plot. Layout adjustments provide the ability to customize the amount of space used by different components of the heat map (dendrograms and image plot)
