# Supplementary figures and images for: A tutorial in displaying mass spectrometry-based proteomic data using heat maps
Source: BMC Bioinformatics. 2012 Nov 5;13(Suppl 16):S10. doi: 10.1186/1471-2105-13-S16-S10 (PMC3489527; doi:10.1186/1471-2105-13-S16-S10)

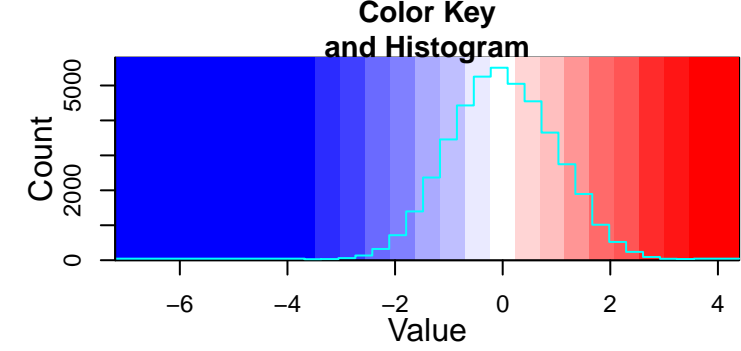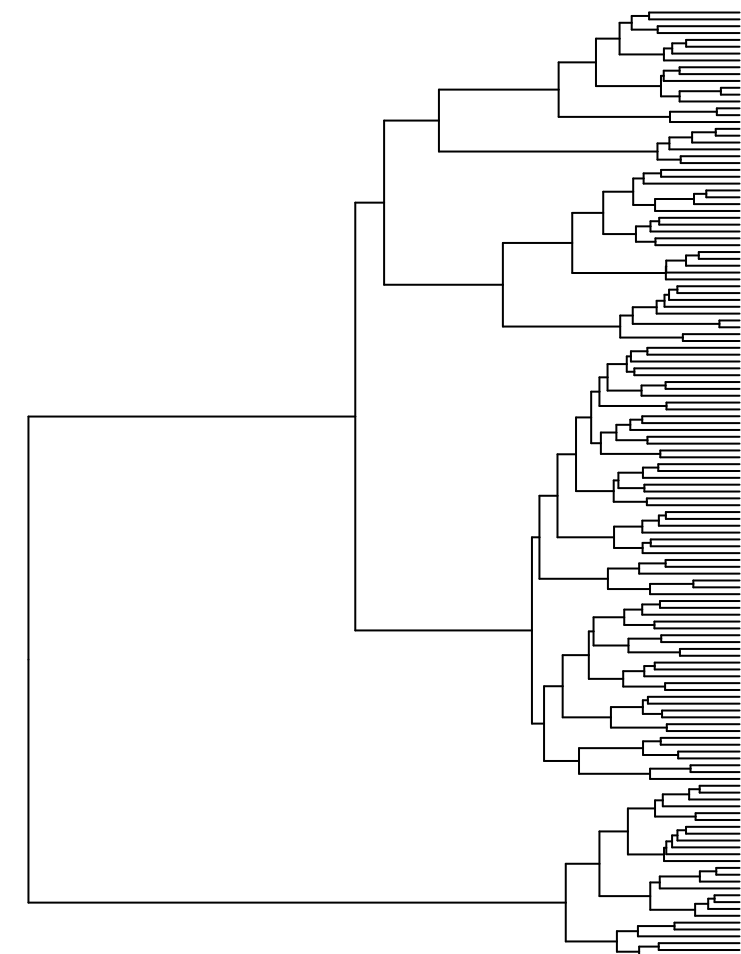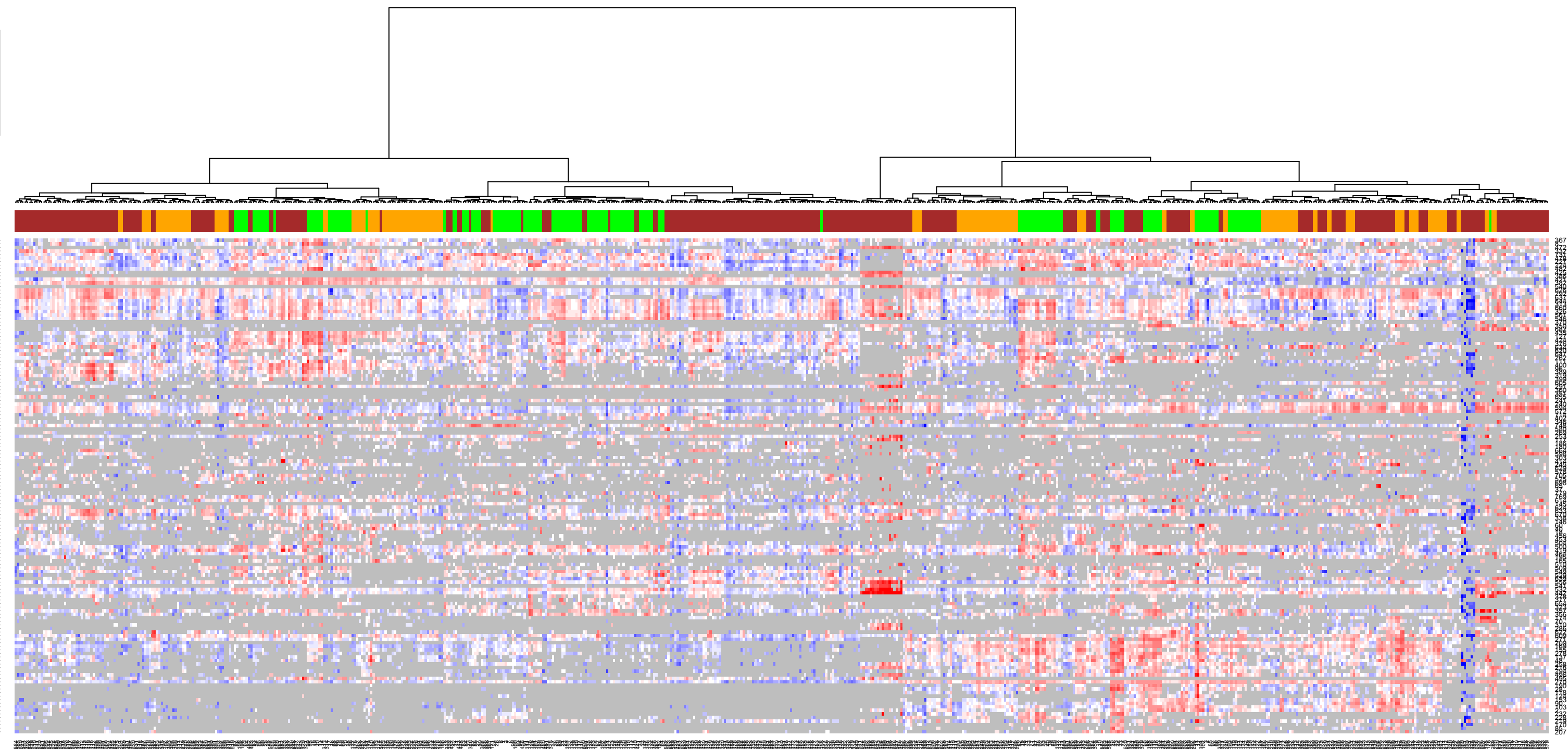

Supplement: Additional file 3 — A larger version of Figure 7. [file 1471-2105-13-S16-S10-S3.pdf]
